# Creating safety amidst chronic contamination: A mixed-method analysis of residents’ experiences in a Southern Italian steel town

**DOI:** 10.1016/j.socscimed.2024.116866

**Published:** 2024-04-16

**Authors:** Maaret Jokela-Pansini, Raffaele Ippolito, Beth Greenhough, Anna Lora-Wainwright

**Affiliations:** School of Geography and the Environment, University of Oxford, South Parks Road, Oxford, OX1 3QY, United Kingdom

## Abstract

This study analyses how residents create safety in Taranto, Italy, a city located next to one of the largest steel plants in Europe. Combining long-term ethnographic research with an online-based survey, our study shows that most respondents recognise and criticise the presence of environmental risks in their daily lives but encounter such risks in complex ways. Contrary to previous scholarship suggesting that pollution can result in alienating residents from their lived environment, this research shows that acute awareness of environmental risks does not necessarily undermine attachment to place but rather can co-exist with or even strengthen it. Our findings propose first that residents *experience and understand* environmental risk mostly through air pollution, but often situate risks outside of their own neighbourhood and inscribe different meanings to such risk. Second, residents *mitigate environmental risk* through practices aimed at creating safety, such as moving away from the industrial area or using everyday practices and reflecting on their responsibility for actions. Third, we argue that residents *create safety* through an attachment and entitlement to place and emotional detachment from pollution and institutional failures. Finally, in line with residents’ concerns about safety and how to secure it, this study embraces a shift in its analytical focus from risk to the quest for safety. By doing so, it provides novel insights into environmental risk perception in industrially polluted areas and reveals the often-contradictory sentiments and practices that such areas invoke in residents.

## 1 Introduction

Due to its location next to the second largest steel plant in Europe, as well as an oil refinery, an industrial port and several landfills, Italian and EU institutions have classified the city of Taranto as a high-risk environmental area. This study analyses how residents in Taranto experience environmental risks in relation to both their health and broader socioeconomic dimensions, and how they mitigate the risks posed by chronic contamination through practices and discourses which create spaces of safety.

This project builds on recent research exploring residents’ understandings of environmental risk, particularly those living in industrially polluted areas. On the one hand, scholars of environmental studies and epidemiology have used quantitative surveys to explore the extent to which residents are unevenly exposed to and perceive different sources of pollution as a risk or threat, how sensitive they are to sensorial impacts such as odours, and how pollution influences (human and non-human) health and individual behaviour (Vigotti et al., 2011; Ban et al., 2017; Cori et al., 2020). On the other hand, ethnographic research in anthropology, geography and environmental humanities has highlighted that people’s everyday experiences of toxic exposure are embedded in unequal social, economic and political structures and power relations, are affected by gender, age, ethnicity, race, socioeconomic background, and place; and are entangled with nonhuman actors and the environment (Pulido, 2017; Davies, 2018; Vasudevan, 2019; Shadaan and Murphy, 2020).

We draw on long-term ethnographic fieldwork and data from an online survey with Taranto residents to examine their experiences in different neighbourhoods, which are exposed to uneven levels of risk. Our aims are to 1) understand how residents experience exposure to environmental risk and 2) how they seek to mitigate it. By doing so, our study will complement epidemiological research on exposure to environmental risk, including environmental pollution, and its clinical effects on their health. The survey data (N = 178) was collected in 2022 through an online survey, which applied a series of open and closed questions and map-based tasks to elicit residents’ experiences of environmental risk. In this context, we understand environmental risk related (but not limited) to improper waste disposal, illegal landfills, car fumes, industrial plants, water, air and soil pollution, mould and humidity (see also Table 2 question number 7). Our study mostly focuses on air pollution, which is highlighted as the key theme by both our study participants as well as literature on environmental risks in industrial areas. We use the term chronic contamination to highlight the temporality of toxic exposure

This article proposes first, that in terms of *experiencing and understanding environmental risks*, the residents often situate these outside of their neighbourhoods, showing greater awareness of pollution at the city level. Second, residents *mitigate environmental risks* by moving away from the industrial area or using everyday practices. Third, residents *create safety* through an attachment and entitlement to place and emotional detachment from pollution and institutional failures.

It is important to note that our original research questions were designed to investigate perceptions of environmental *risk*. However, our research findings suggested that participants spoke about imagining and creating *safety*. Hence, we shifted our analytical focus to better represent our participants’ priorities. In environmental research, the term safety often refers to practices that prevent or reduce exposure to environmental risk/contamination. It is mainly associated with policy-oriented research and studies of the measures taken by governments, industries and employees to enhance individual and environmental health (Patwary,O’Hare and Sarker, 2011). By contrast, this article illustrates the affective dimensions of residents’ efforts to create safety through everyday discourses and practices. Here, creating safety means fostering and celebrating connectedness to the place and acting upon what are understood to be the best ways to protect family members. We argue that focusing on safety counters simplistic approaches to risk awareness as inevitably leading to residents either leaving the contaminated place, or staying because they do not understand or simply ignore such risks.

In the following, we first provide background on Taranto’s past and present as a steel town and chronically contaminated area, and then situate our research within wider scholarship on environmental risks. We then present our methods (ethnographic research and online survey) and discuss our three key findings (experiencing and understanding environmental risk, mitigating environmental risk, and creating safety) before offering some conclusions.

## 2 Background: Taranto, a chronically contaminated area

Taranto is one of the main industrial areas in the Apulian region. The industrial park includes Europe’s second-largest steel factory, a petrochemical plant, an industrial port, and various industrial landfills. The industrial park is responsible for heavy emissions containing a mixture of highly carcinogenic pollutants, including dioxins, PCBs, benzo(a) pyrene, benzene, heavy metals and PM10. Dioxin emissions alone, for example, account for 80% and 8,8% of Italian and EU total emissions respectively (Mangia et al., 2013). Epidemiological studies have associated these emissions with chronic degenerative diseases ranging from cardiovascular and respiratory pathologies to various forms of cancer which result in excess mortality higher than those of the entire Apulia Region (Palmisani et al., 2020) and that also affect young adults and children (Pirastu et al., 2013; Bansal et al., 2019). These include genetic mutations of the foetus during pregnancy, which result in the development of serious neurodevelopmental pathologies (Lucchini, 2019; Renzetti et al., 2021). Taranto’s chronic environmental contamination has also caused considerable economic damage to the pre-existent productive activities in the neighbouring areas. For example, pollution is responsible for the destruction of mussel farming in the first inlet of the Mar Piccolo (Small Sea), the culling of thousands of cattle belonging to dairy farmers due to their being exposed via pasture in highly polluted areas (at a radius of 20 km of the steel plant) and other forms of severe damage to the food industry (Vigotti et al., 2011).

The steel plant, which has operated since the 1960s, has been the focus of particular controversy. The factory is also known as ex-ILVA due to its first owner, the ILVA group, and it has been operated since 2017 by Arcerol Mittal, the largest steel producer globally. It has been the target of a series of interventions from environmental justice movements which have successfully appealed to the European Court of Human Rights, arguing the levels of pollution emitted by the factory constituted a violation of articles 2, 8 and 13 of the European Convention (Cordella Et Autres c. Italie, 2019). More recently, an appeal to the United Nations Office of the High Commissioner for Human Rights classified Taranto as a “sacrifice zone”, thus re-inscribing it as a high-risk area (UN OHCHR, 2022). These actions have led to some efforts from the local government to force the factory to suspend polluting activities. However, in light of the plant’s strategic importance for the Italian industry, the Italian Government has repeatedly intervened to try and keep the factory open (Palmiotti, 2023), approving over 15 ad-hoc laws to allow the factory to operate despite its repeated national and international legal violations.

Several environmental and community groups, including *Giustizia* per *Taranto* and *Genitori Tarantini*, have lobbied for the steel plant’s seizure. Activism range from citizen science initiatives to monitor and mitigate pollution (Alliegro, 2020), to coalitions to link labour and environmental struggles around the primacy of reproduction (Barca and Leonardi, 2018), to public debates between industrialists and environmentalists through technoscientific language (Greco and Bagnardi 2018), to more subtle everyday practices of pollution mitigation and future-making among young people in Taranto (Jokela-Pansini, 2022; Jokela-Pansini and Militz, 2022) and place-making through cultural and creative working practices (Coppola and d’Ovidio, 2018). Conversely, other studies document a widespread sense of defeat and resignation among residents who do not participate in environmental advocacy (Ippolito, 2022). This article zooms in on residents’ everyday experiences by illustrating how the entanglements between perceptions of risk and attachment to Taranto result in residents’ everyday efforts to create safety amidst chronic contamination.

## 3 Literature review and theoretical framework

This study builds upon scholarship in geography, epidemiology, environmental studies, and related fields exploring residents’ experiences of environmental risks, particularly regarding epidemiological health effects and unequal distribution of exposure, and daily lived experiences of pollution and critical understandings of contamination.

Living close to industrial production sites and other sources of environmental pollution generally means a higher likelihood of contamination and thus, poorer health outcomes (Amram et al., 2011; Jephcote and Chen, 2013). Epidemiological studies highlight how environmental harm is disproportionately distributed among socially deprived communities (Di Fonzo, Fabri and Pasetto, 2022). Environmental justice scholars have similarly emphasised that environmental inequalities reinforce and, at the same time, reflect intersecting social hierarchies and environmental racism. These studies highlight how people of colour are disproportionately bearing the burden of industrial pollution (Holifield, 2001; Pulido, 2017). Despite national and international environmental regulations, some communities get ‘dumped on’ while others escape (Bullard, 2000). Scholars have demonstrated that in European countries including France, Hungary and Slovakia, towns with higher proportions of immigrants and ethnic minorities are more likely to have a variety of hazardous sites and to host greater numbers of sites (Laurian, 2008; Harper, Steger and Filcák, 2009).

Residents’ experiences of environmental risks and responses to risks are key themes in epidemiological and environmental justice literature. Existing survey-based research on residents’ experiences, particularly of air pollution, has focused on residents’ understandings of sensorial perceptions (De Feo, De Gisi and Williams, 2013), social and psychological effects (Bush, Moffatt and Dunn, 2001; Barnes et al 2002, access to and trust in environmental information (Coi et al., 2016), cerand the influence of social status and capital on the likelihood of residents regarding pollution as a threat (Bickerstaff and Walker, 2001; Wakefield et al., 2001; Muindi et al., 2014). Other work has focused on resident’s attempts to mitigate the effects of pollution, including changes in behaviour (Ban et al., 2017) such as reducing outdoor activities (Giles, 2014), enhancing indoor protection (Semenza et al., 2008), or driving instead of walking/cycling (Elias and Shiftan, 2012). Altogether, survey-based studies suggest that risk is negotiated at the intersection of epidemiological, social, economic, and political perceptions within chronically contaminated communities, but with the assumption that place is experienced only as a site of risk and harm.

Similarly, ethnographic studies of chronic contamination have examined the temporality of toxicity (Davies, 2018), contestations over what counts as evidence of environmental health harm (Checker, 2007; Lora-Wainwright, 2013), the bureaucratic and legal hurdles to proving such harm (Fortun, 2001), gaining biological citizenship as residents of contaminated areas (Petryna, 2004) and the production and use of scientific knowledge and counter-expertise on toxicants (Allen, 2018). Concerns with contamination highlight the influence of toxic chemicals, substances, and situations across generations and the role of high-tech laboratories as pathways for knowing and mitigating exposure (Wahlberg, 2018; Lamoreaux, 2021). These studies illustrate how the experiences of contamination, the technoscientific apparatus which measures it, and the strategies embraced to mitigate it, are shaped by their socio-cultural contexts.

Research to date has further highlighted the importance of considering the material, psychological and social dimensions of chronic contamination experiences (Agovino, Cerciello and Musella, 2021; Sullivan et al., 2021). Pollution at the community level can result in worry and anxiety, which in turn require a holistic approach to be understood and addressed (ibid.). If ignored by institutions and delegitimised, the specific understandings and meanings communities place on pollution can perpetrate psycho-social harm (Sullivan et al., 2021). Such harm can culminate in a sense of loss of community and even a loss of home. Edelstein’s monograph on ‘contaminated communities’ in Legler, New York State (Edelstein, 1988), illustrated how pollution can alter residents’ attachment and even cause an ‘inversion of the sense of home’ whereby home is no longer safe (1988: 48-9). Conversely, communities affected by chronic contamination may opt to avoid dwelling on its effects (Lou, 2022) and focus on ‘wagering life’ (Valdivia, 2018), especially when such communities are marginalised and feel powerless (Lora-Wainwright 2021).

At the same time, these studies have demonstrated that attachment to home is connected to the relationships, history, and heritage of the place the people inhabit. We thus situate our study within a larger scholarship on the past, present, and future of place and place-making (Massey, 1995; Pierce, Martin and Murphy, 2010). In the context of Southern Italy and Southern Europe more broadly, scholars have highlighted that such place-making is connected to the effects of both past and present diverse political and economic crises but also, cultural richness and beauty (Knight and Stewart, 2016; Pipyrou, 2016; Iovino, Cesaretti and Past, 2018; Muehlebach, 2020).

We build on this extensive body of research, which highlights both exposure to and perceptions of environmental risk. For our analysis, this stimulates a critical reflection on the relationship between belonging and exposure and highlights individuals’ and communities’ narratives and practices regarding contamination and risk, but we also extend such analyses through focusing on how perceptions of risk and (in)actions undertaken to respond to and mitigate risk, are further complemented by other discourses and practices which seek to recognise and create safety.

## 4 Methods

Since 2019 two of the co-authors have conducted long-term ethnographic fieldwork in Taranto, including over 80 interviews with activists, health professionals, artists, and other residents, mostly in the neighbourhoods close to the steel factory (Tamburi, Paolo VI, and the old town). We have analysed some of the findings in previous papers (Ippolito 2022, 2023, Jokela-Pansini and Militz 2022 and Jokela-Pansini 2021, 2022). This article draws on mixed methods, including both our previous and ongoing ethnographic research and an online survey (2021-22) which employed a series of open and closed questions and map-based tasks to elicit residents’ experiences of pollution. Previously collected ethnographic data was vital for developing the survey questions, and informed how survey data was subsequently analysed and interpreted.

### 4.1 Ethnographic research

The first author conducted fieldwork for nine months in Taranto between 2019 and 2021. Between 2020 and 2022, the second author conducted 13 months of fieldwork in Taranto, Italy, being from the city themself; They were also involved in environmental advocacy with several key organisations in Taranto. Both authors focused on residents’ different experiences of living in Taranto, particularly the ways the presence of the steel plant and the industrial pollution affected their everyday lives, and their responses to these.

### 4.2 Online survey

The survey of Taranto residents we conducted had three aims: to understand 1) how residents in different neighbourhoods experience their health in relation to environmental risks spatially, materially, and temporally, 2) how they mitigate such risks, and 3) how these risks shape their understanding of individual and collective wellbeing. We employed the online software Maptionnaire, which allows for the integration of both conventional survey questions as well as visual prompts and interactive maps.

Drawing on our ethnographic experience, we designed the survey around four categories of residents’ experiences with pollution: 1) Spatial and material perceptions of environmental risks, 2) Experiences of health in relation to environmental risks, 3) Mitigating environmental risks, and 4) Acting on environmental risks. Our survey consisted of 22 questions: closed questions, including matrix questions using Likert-Scale from 1 to 6, multiple-choice questions allowing respondents to choose either one or several options, as well as open questions. All closed questions contained an open-answer field to allow the respondents to communicate their own experiences and practices (Silverman, 2010). The survey was conducted from November 2021 to January 2022, with a sample of 178 respondents over the age of 18. The demographic categories were drawn from the Italian National Institute of Statistics Istat (istat.it).

Table 1 summarises the key sociodemographic characteristics of the respondents. Around half of the respondents (52%) are female, and the majority (61%) are 35–64 years old. 46 per cent are employed, and over half of the respondents report their income at less than 20 000 euros per year. Particularly notable are the consistently low levels of income across all respondents and fairly high levels of unemployment, characteristics that have been observed both in relation to the Apulian region and Italy more widely (Cacciapaglia, 2023).

One of the challenges of using an online survey tool was the uneven access to the internet and/or limited technological abilities amongst some target participants, particularly among the elderly. To compensate for this, two research assistants, Delia De Marco and Jlenia Mancino, conducted additional face-to-face surveys with residents over 65 and with residents living in more marginalised neighbourhoods such as Salinella and Tamburi. Their deep knowledge of local groups and neighbourhoods enabled us to reach out to communities and residents whose voices would not normally feature in accounts of Taranto’s pollution. The study was approved by [removed for anonymity], and the participants were informed about data privacy and gave their consent to use the data at the beginning of the survey.

**Fig. 1:**
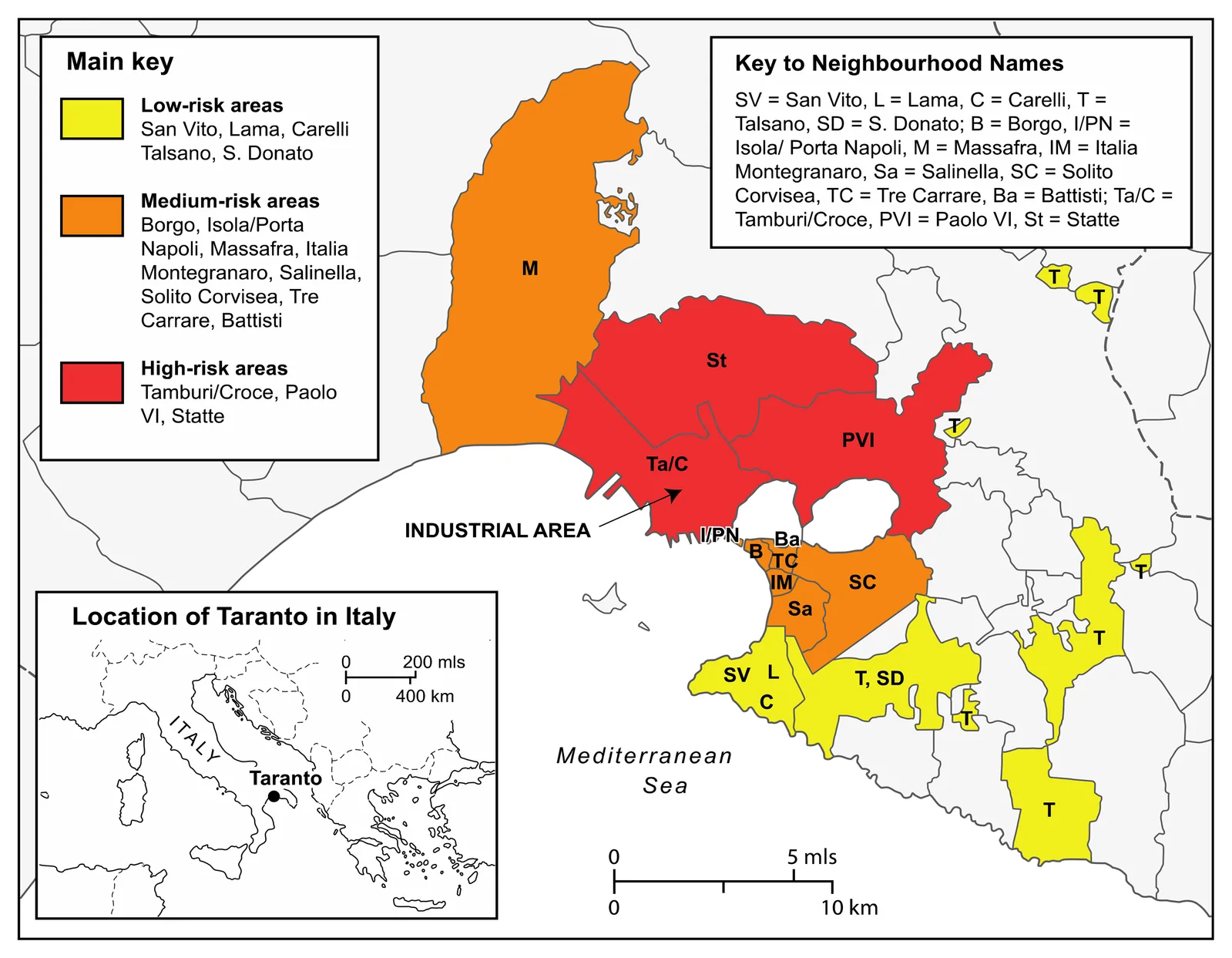
Taranto neighbourhoods.

**Fig. 2:**
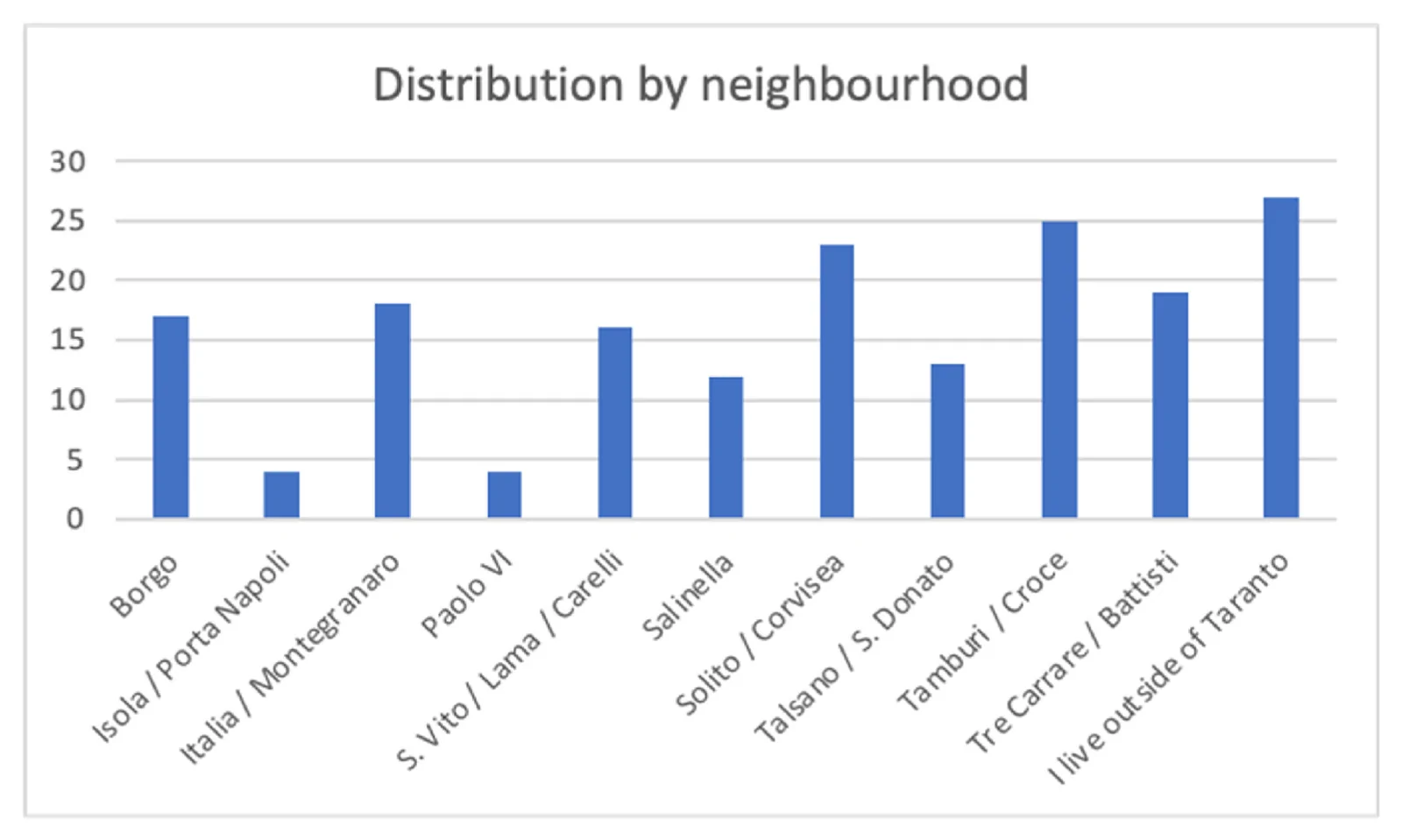
Distribution of respondents by neighbourhood.

For analytical purposes, we categorised neighbourhoods on the basis of existing epidemiological studies (Mataloni et al., 2012; Leogrande et al., 2019), so that those like Tamburi and Paolo VI, which are closest to the industrial area, are classified as high risk, those in central Taranto but not closest to the industrial area are classified as medium risk and the neighbourhoods which are further away are coded low risk (Fig. 1). We received responses across all neighbourhoods (see Fig. 2). Descriptive statistics were generated to offer a broad overview of emerging trends in the closed and multiple-choice questions. We used content analysis to analyse the data from the open questions and focused on the way respondents’ perceptions of pollution were contextual and situated, paying particular attention to anomalies, inconsistencies, and unexpected findings in relation to existing studies. In other words, we were interested in where the respondents were speaking from, in addition to what they said (see Table 2).

Through content analysis, we identified a number of recurring themes such as mobility and moving away, seeing the closure of the factory as a solution to mitigate pollution, or talking about Taranto with affection/ attachment to place. These served as initial categories for organising the analysis and were expanded and refined through multiple readings of the data set. Ultimately, three recurring themes emerged from our content analysis as most useful for organising the discussion of our findings: experiencing and understanding environmental risk (5.1), mitigating environmental risk (5.2) and creating safety (5.3). Although the first two themes correspond to the survey design, their centrality was validated by respondents’ detailed reflections on the materiality and spatiality of pollution (particularly air pollution) and on strategies for mitigating risk, whether the question centred around health effects, environmental problems, or mitigation strategies. The third theme, creating safety, emerged from the frequent comments participant’s made about attachment to place, which for many was stronger than the fear of pollution.

## 5 Findings and discussion

### 5.1 Experiencing and understanding environmental risk

#### 5.1.1 Situating and understanding risk

**Fig. 3:**
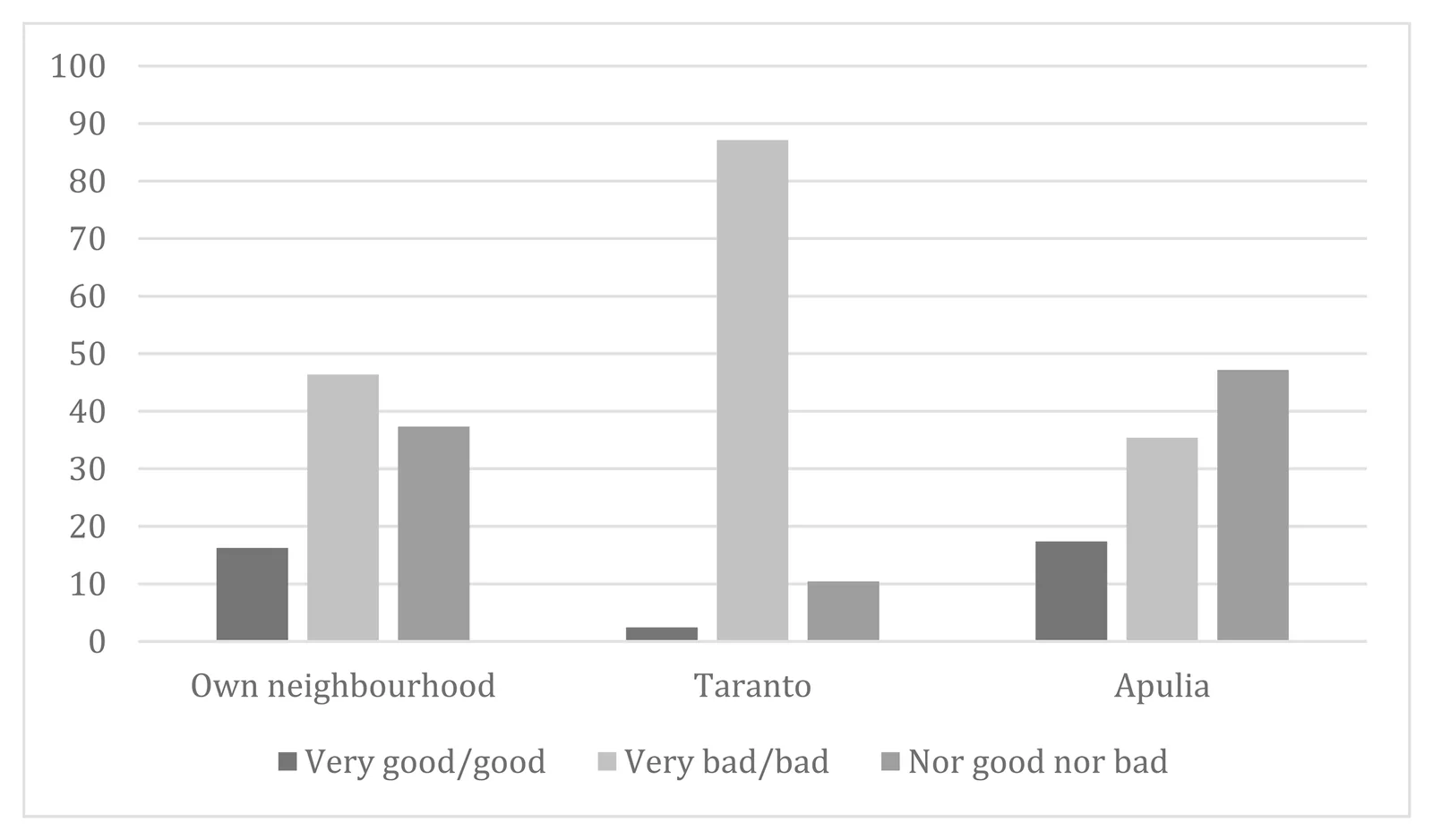
Respondents’ own assessment of the environmental situation by location.

Most of the survey respondents classified the environmental situation in Taranto city generally as “bad” (Fig. 3). Significantly, however, around half of the respondents felt that the environmental situation was bad in their own neighbourhood and around one-third felt this way about the region, Apulia. The graph below suggests residents tend to situate risk in other areas more than their own.

Residents in the most heavily polluted neighbourhoods (Tamburi particularly) were more likely to identify environmental risks in their neighbourhood than those who lived further from the industrial complex. However, they also situated these complaints within a broader dissatisfaction with the local environment and socio-economic inequalities, and they regarded pollution in Taranto at large to also be severe. This results in the neighbourhood coming to be regarded as a safe place as opposed to the city itself (except for the neighbourhood closest to the factory, Tamburi).

**Fig. 4:**
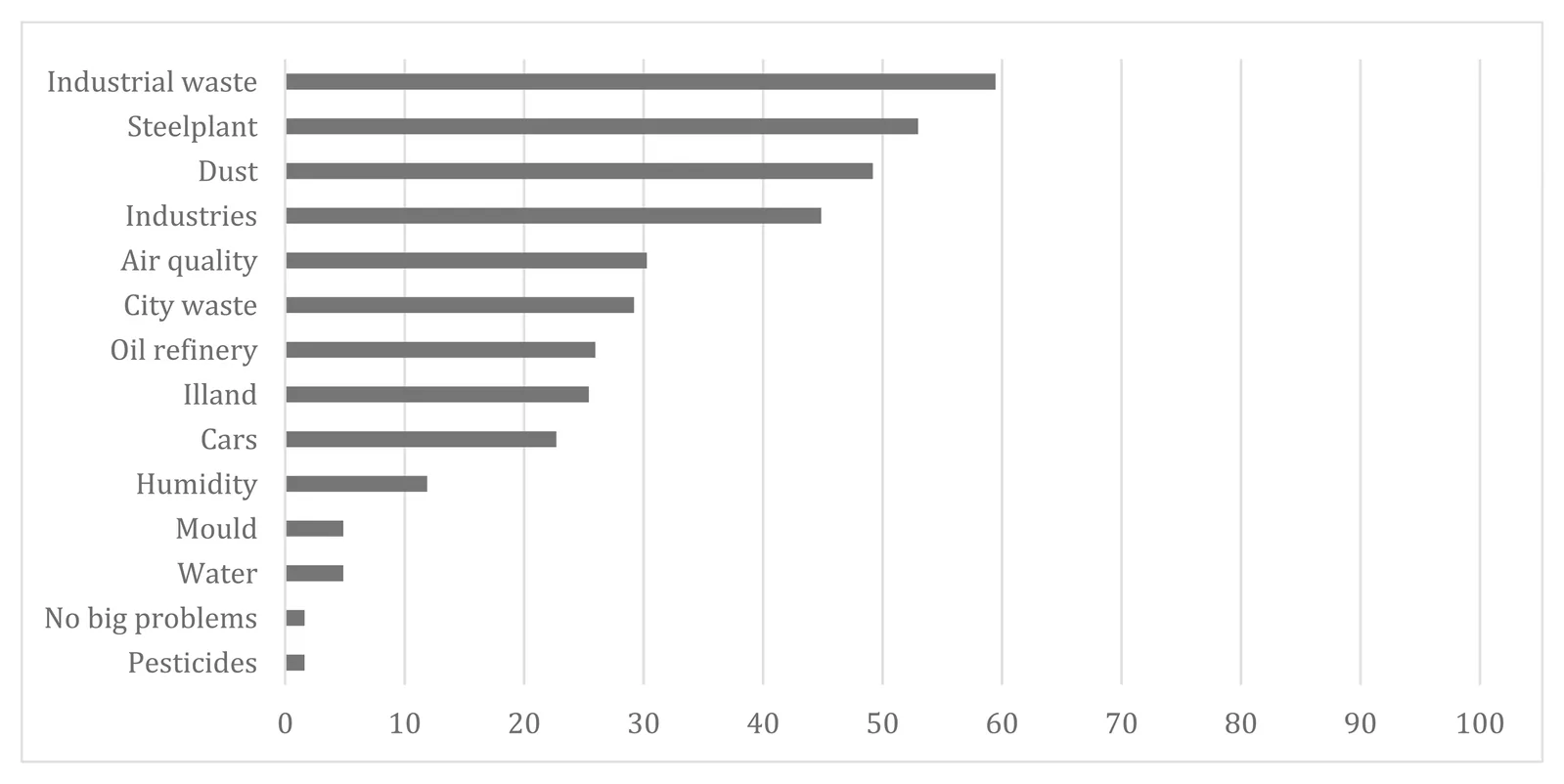
Respondents’ assessment of environmental problems by type of problem, % (n = 185) Note: Respondents were allowed to choose maximum 3 out of 14 options.

Industrial waste and the steel plant were reported as the most significant environmental problems in the respondents’ neighbourhood (Fig. 4). The open-ended responses revealed that there was a discrepancy between those in higher and lower-risk areas in terms of what environmental problems they chose to emphasise. Residents of **lower-risk areas** reported issues such as traffic, waste disposal (lack of bins, lack of adequate collection, poor disposal by citizens, general dirt, and degradation), the poor condition of the local roads (road surface, flooding) and the lack of green space or poor care for it. A smaller number also raised concerns about dog excrement, parking, unemployment, and crime. While air pollution (particularly from the factory) was mentioned across neighbourhoods, most respondents in the highrisk areas highlighted air pollution specifically, declaring that the air is ‘unbreathable’ and ‘unliveable’ and noting that they need to keep windows closed at all times as a consequence. This is strikingly echoed by the comment from two respondents: *We die here* (si muore), while another noted that *people here get sick and have no means to be treated*. Accordingly, residents of high-risk areas highlighted that improving the local environment, particularly air pollution, is their most important priority.

However, as the quote “people here get sick and have no means to be treated” shows, concerns with pollution are inseparable from broader challenges such as access to healthcare. Indeed, even residents of high-risk neighbourhoods reported other risks alongside pollution, such as poor infrastructure, problems with individual waste disposal, low levels of education, widespread corruption, and crime. Across all neighbourhoods, respondents stressed the importance of improving services, especially healthcare, education/civility (referring to both formal education but also to corruption and social degradation) and decreasing crime rates. Such a diverse range of intersecting concerns highlights that pollution is but one of numerous challenges for residents of polluted areas, who experience multiple forms of environmental, social and political exclusion and harm (Lora-Wainwright, 2013; Ippolito, 2022; Jokela-Pansini, 2022).

#### 5.1.2 Experiencing air pollution

Across all survey respondents, there was a strong awareness of air pollution and the harm it causes, both to themselves and others. This has earned the steel industry the local nickname, “il mostro” (the monster), which many of the participants used in their responses. The materiality of pollution was often central to the evidence provided, for instance, through references to “fine dust and heavy metals” and comments on how the wind spreads dust. One stated: “*the closer you get to the city, the more you* ***feel*** *the proximity of ilva”*. Respondents also reflected on the impact of wind upon the distribution of pollution: winds blowing from the South-East cause distress to the local population due to the increased temperature and humidity they are associated with; cooler winds from the North-West result in a visible increase of pollution in the areas neighbouring the factory (89). On these days, public health authorities issue so-called ‘wind days’ warnings, advising the local population to stay indoors and keep children away from outdoor areas. Wind may also spread pollution well beyond the high-risk area, and some who lived as far as 15 km away commented that “dust from the industry” can still be found in their home (102). Others argued the wind could also help clean the city from dust: “Because we often have ‘scirocco’ (wind from Sahara), we are fortunate to send possible dust towards the west.”

Perhaps the most poignant illustration of the experience of environmental risk is the use of the word “**poison**” (veleni - 55) to refer to pollution and residents’ acute **concerns with illnesses** caused by pollution. Leaving the city and coming back can serve to alert residents of the harmful effects of pollution: “I lived away from Taranto, and I was well. After coming back, a few months later, I became allergic to lots of things, and my skin changed radically” (12). One woman reported having breastmilk tested, and the dioxin level was higher than elsewhere (53). In one case, ill health resulting from pollution was described as “an infringement on human rights” (16); others noted that “the harm of pollution on health is still little understood and […] it can affect mood, anger, low enthusiasm and self-esteem, and cognitive skills” (12) and that cancer incidence is higher than elsewhere (137), which reflects many respondents’ awareness of existing epidemiological studies.

### 5.2 Mitigating risk

Efforts to mitigate risk unfold through a spectrum of practices, varying from the desire to move away (5.2.1. mobility) to a range of strategies involved in mitigating damage to one’s health and the health of their family members (5.2.2. everyday practices). Most respondents found that in the end, such practices were hopeless, and the only way to reduce pollution was to close the steel factory (see 5.2.3. The futility of mitigation efforts).

#### 5.2.1 Mobility

Similarly to previous findings (Jokela-Pansini and Militz 2022), residents perceived moving as one strategy to protect themselves and their family, implying a strong sense that living near the factory is harmful. Moving away or changing the neighbourhood was mentioned to answer questions about mitigating pollution (11), in addition to answering the direct question about moving (13). 39 per cent of the respondents had already considered moving away from Taranto, and 31 per cent had considered changing the neighbourhood. One observed: “We all moved to Rome and Padua. The only one left behind was my father, who, in fact, has developed skin cancer” (36; see also 34, 35, 137). This not only suggests that moving was a direct response to avoid pollution but that remaining in Taranto resulted in illness. Some respondents did not move to a different city, but to what they perceived was a cleaner neighbourhood or area, further from the industry, particularly in the countryside or close to the sea. For instance, one replied to the question “Are there any other things you do to protect you and your family from pollution?”, “we live in the neighbourhood on the other side of the city from the industrial area” (86, see also 102). Many of the respondents referred to physical distance in kilometres to define whether they felt exposed to pollution. For instance: “I bought a house as far away as possible from ILVA after living 30 years practically next to it”, or, “I made a choice six years ago to move to the countryside (also in the province of Taranto) to breathe and eat well and healthy, about 40 km away from the city.”

Notably, the decision or the wish to move was expressed as a form of care for family members: “Whoever has children has a moral duty to move … it costs me a big sacrifice in terms of money and time, but I do it because I would not live well otherwise” (21) “I think of this (moving) often, for me and for my mother” (35), “you can’t raise children in a city where the likelihood of developing cancer is 400% higher than in the rest of Italy, this is not life, it’s survival” (137). “I moved 30 km away from the steel plant to protect my son’s life as much as possible. I still work a few miles from the steel plant (I have a family store and unfortunately commute). But at least my son sleeps safer in the countryside. It changes little for me now.” This final quote elucidates an awareness that, in some cases, the mitigation through moving one’s family home away from pollution may not protect family members who continue to work in its proximity but reiterates the moral aspects of moving as a form of care for more vulnerable family members.

A number of respondents expressed a wish to move (for instance “to the mountains, to less contaminated regions” 80) and a sense of regret that their current practical circumstances did not make this possible: “I can’t do it, if I was 20 years younger I wouldn’t think twice” (84), “I think about it (moving) constantly but I can’t for family reasons” (147), “we don’t have the financial conditions to move” (142), “my husband works here on short contracts and I could not get a mortgage (elsewhere)” (152) “we cannot sell our devalued homes otherwise I would run away like a thief in the night” (151). This phrase begins to articulate some of the mixed feelings which surround moving on the one hand and attachment to Taranto on the other. “Running away like a thief in the night” implies, at the very least, feelings of betrayal and guilt surrounding decision to leave Taranto. (We return to this point below).

#### 5.2.2 Everyday practices

**Fig. 5:**
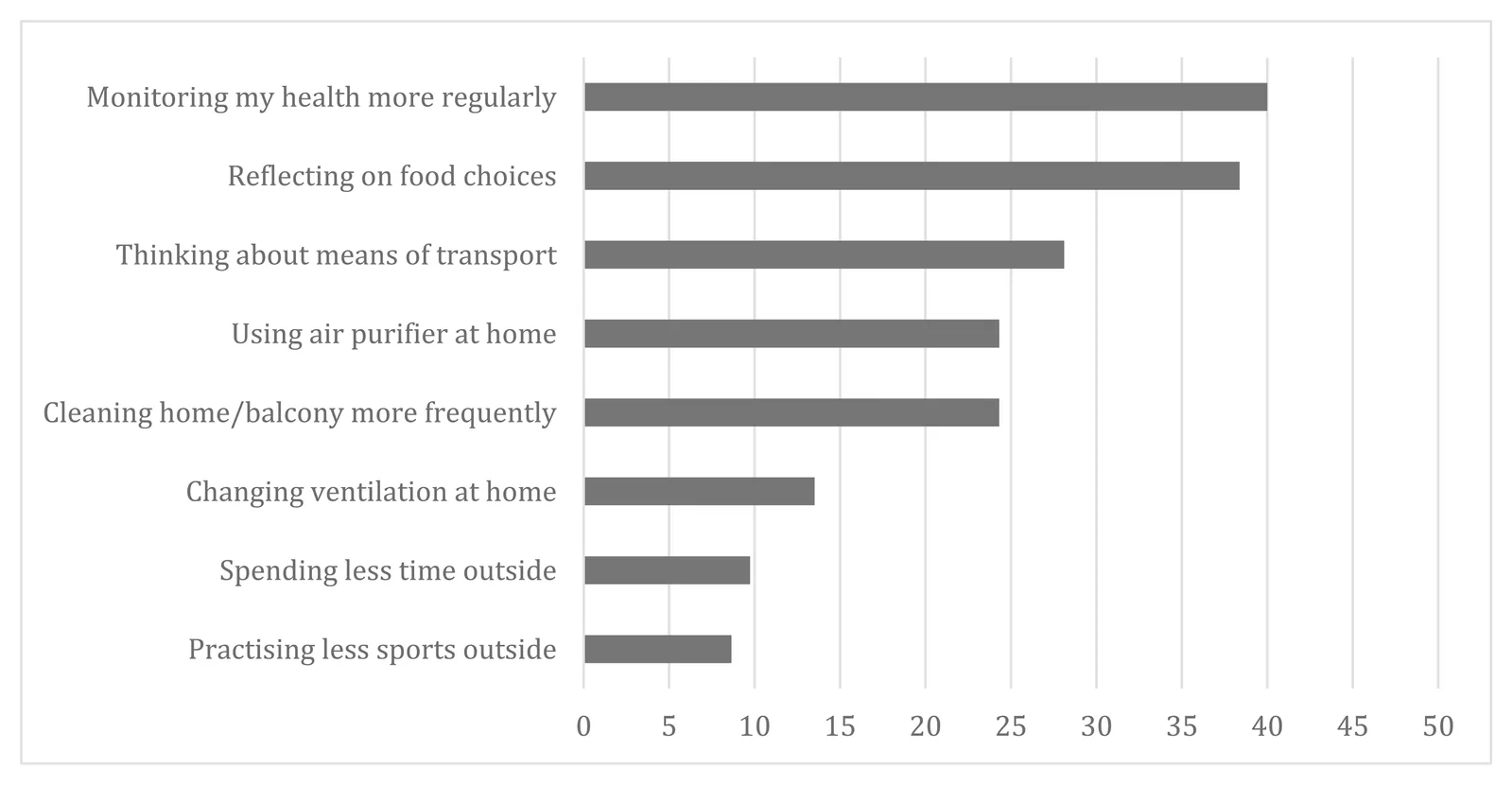
Respondents’ practices to mitigate exposure to pollution, % (n = 185).

Most respondents, especially from high-risk areas, also described individual strategies to minimise exposure and protect their bodies and those of their closest family members (see Fig. 5). The most frequent measures included monitoring own health more regularly (40%) and reflecting on food choices (38%). In the most exposed neighbourhood, Tamburi, residents referred to doing less sports, avoiding staying outside (137, one specified that children in particular should avoid staying outside, 147), cleaning frequently everywhere inside the house and not using outside shoes inside the house (144); another noted: “I haven’t taken my shoes into the house since my son was born 38 years ago” (145). In other neighbourhoods, residents mentioned “prevention, including annual breast check-ups” (170), use air purifiers at home, avoiding foods grown illegally or in high-risk areas, and driving less. These statements reflect many studies on environmental risks describing individual practices as an attempt to cope with the impacts of pollution. This places the emphasis on resident’s resilience (as opposed to, for example, the state’s obligations). In other words, these actions are often not targeted at changing the system (though they can be) but rather at existing in it (and sometimes challenging it) ethically and through everyday practices (Liboiron, Tironi and Calvillo, 2018; Tironi, 2018; Valdivia, 2018).

Respondents highlighted the need for individuals to “do something” and take more responsibility as a practice to mitigate environmental risks. On the one hand, respondents argued that any problem requires everyone’s commitment: “It is everyone’s duty to do something to improve [the environment]. Of course, there are big problems, too; but each one of us should do something” (120). Another respondent suggested that problems require “the attention of every individual every day, because some bigger things do not depend on us” (104). Both these statements illustrate that individual responsibility is presented as a response to powerlessness in the face of bigger forces—an effort to reclaim agency. It is sometimes proposed as a palliative, given the remote likelihood that industries will close: “ILVA is unlikely to close, people need to be responsible, starting from the smallest units” (53). But the importance of individual responsibility is also expressed across several answers as a need to “educate citizens” (78–92). In other words, individual responsibility can only be enacted once citizens have access to better education. These statements suggest a strong wish to play their part and to imagine Taranto as a safe place to live, but they also suggest that some systemic changes are required.

The acute awareness that pollution is harmful and widespread led many to conclude that mitigating pollution was impossible (see 130, 132, 151, 171), even for those who lived in lower-risk neighbourhoods: “although we are 10 km away, when the wind is strong the dust reaches my area very visibly as well, and even in the sea.” This sentiment is confirmed by the high incidence of the statement that to improve the quality of life in the city, the steel plant should be closed (e.g. 154). Respondents stated that the measures the company was taking, for example, to reduce the distribution of minerals through the air, were not enough: “The large sheds built to cover the mineral parks are somewhat like large carpets under which to ‘cover the dust’” (102).

### 5.3 Creating safety

#### 5.3.1 Attachment and entitlement: “this is our city”

Strikingly, the frustrations and acute awareness about pollution’s damage to health, which are detailed in the previous subsection (5.1), often do not result in a wish to move away. A strong sense of attachment to the city of Taranto is apparent across several of the open-ended survey responses, in relation to questions related to the local environment, and across ethnographic data collected by two of the authors. Such attachment to Taranto is put forward as a reason for residents to refuse to move even when they know pollution is severe; or to move only to the periphery but not outside Taranto province. The moral overtone of such emotional attachment is clear in statements such as: “it [speaking negatively about Taranto] would be like bad-talking your mother” or “it is unfair to abandon one’s city” (16). The strongest formulation, which appeared a number of times, put it plainly in terms of ownership: “this is *our* city, they should leave” (33, see also 76, 83, 155, 157).

These statements highlight a sense of entitlement, not merely attachment, which intersects with a profound pride in Taranto’s history, cultural and culinary heritage, and beautiful scenery (83) (see also Muehlebach, 2020). Many expressed their wish for more investment in the region focusing on tourism, protecting the environment, fighting crime and tackling unemployment through targeted interventions enabling young generations to stay (60, see also 83 and 147; see also Greco and Di Fabbio, 2014; d’Ovidio, 2021)). In one particularly poignant response, the privileging of “industrial interests” and the failure to “guarantee the right to health and psycho-physical wellbeing to residents” is described as a “human rights violation” (16). By expressing such profound belonging and entitlement to living in Taranto, residents also envisioned it as a desirable place to live, despite the current pollution levels, which reflects other ethnographic studies on industrial areas (Valdivia, 2018; Vasudevan, 2019).

#### 5.3.2 Emotional detachment from pollution and institutional failures

In addition to creating safety through an attachment and entitlement to the city, the respondents create safety through an emotional *de*tachment from pollution and institutional failures. When asked, ‘Who do you think is responsible for mitigating pollution?’, the respondents expressed their dissatisfaction with a range of overlapping environmental, social, and political shortcomings (ibid.), particularly institutional negligence and the feeling of having been abandoned by local, national, and European authorities alike. “If the European Parliament began addressing Taranto’s situation, more leverage could be brought on national governments that have only served the industry interests, without improving the local economic conditions”.

Answers to the question ‘How do you think environmental pollution could be reduced?’ reflected the residents’ frustration with local and national authorities. They complained that the environmental health plight (and Tamburi neighbourhood in particular) is seen as secondary in the pursuit of the national industrial project and that their welfare is wilfully neglected by corrupt institutions. The respondents’ lack of trust pervades not only attitudes to local and national authorities whose duty it should be to safeguard their welfare but also to organisations that are set up with the explicit aim of countering pollution.

The respondents’ frustration is manifested in comments suggesting that the industry will never close, that pollution cannot be reduced for as long as the industry operates and that the effects on locals’ health can scarcely be reduced. Discursive emotional detachment allows resentment, powerlessness and resignation to co-exist with emotional attachment to Taranto; it makes systemically produced suffering less unbearable and allows feelings of safety to emerge despite pollution. These discourses serve as a way for individuals to reassure themselves that despite using various practices to mitigate pollution, tackling the industrial “monster” is not their responsibility, or at least not their responsibility alone.

The prevalence of everyday practices for mitigating harm described in survey responses (5.2.), on the one hand, confirms that residents care for themselves and their families and manifest such care through practical interventions. On the other hand, discursive emotional detachment is another way of creating distance between residents and pollution when physically distancing themselves from the steel plant is impossible. Such discourses nurture the imagination of a safe place where pollution is less prominent while making practical interventions to minimise its harm.

## 6 Conclusions

In this study, we draw on ethnographic research and survey data to understand the lived experiences of some of Taranto’s most marginalised residents amidst chronic contamination. From our ethnographic and survey data, we drew out two conclusions: 1) the seeming contradiction between strong awareness of exposure to pollution and discourses of safety and 2) the negotiation of physical and emotional distancing from the factory.

Firstly, our data highlighted that Taranto residents are acutely aware of the harmful effects of pollution: they reflect on its materiality (dust, in particular) and its effects on health and the surrounding environment. Notably, however, participants often experienced their own neighbourhood as less polluted than the city itself. We argue that this enables a feeling of belonging, which is attached to feeling safe to persist (see also Yuval-Davis, 2006). Our study, therefore, contributes to understanding how experiences of environmental risk shape emergent environmental health subjectivities (see also Lora-Wainwright, 2013). Our findings demonstrate that polluted neighbourhoods are experienced as sites of exposure but also safety. Contrary to Edelstein’s (1988) work on residents’ experiences with pollution in Legler, this study showed that feelings of attachment to one’s home and one’s city can endure despite experiences of toxicity. While some studies have depicted residents’ care practices as a way to connect with and take care of the chemically polluted environment (Lyons, 2018; Tironi, 2018), we found that in Taranto, participants’ attachment to place resulted in everyday practices and discourses aiming at creating safety.

Creating safety amidst chronic contamination offers a counter-narrative to often more prominent visions for Taranto’s future found in epidemiological studies or official narratives. Such attachments allow residents to see beyond pollution and to emphasise other qualities their place has to offer – including beautiful architecture, a sense of community, history, culture, and a seaside location. In doing so they create counter-narratives of their city and defy the transformation of its cultural richness into misery (Ceronetti, 1985; quoted in Iovino et al., 2018). This point is fundamental for interpreting other data about people’s perceptions of environmental risks, how these may be impacted by attachments to particular places, and a reluctance to characterise them merely as sites of environmental degradation (Ippolito 2024).

Secondly, some residents responded to these experiences by physically distancing themselves from the factory, relocating and/or often using measures in kilometres to emphasise a sense of safety from pollution. Thus, these experiences of safety are to be understood in relation to the proximity of the steel factory. Moving away or living in the countryside were often referred to as possible solutions for improving health and quality of life. In some ways, this follows long precedents of a desire to “escape to the countryside” (Elson and Shirley, 2017, 2017), but such movements also reflect how environmental pollution is a form of dispossession that forces people to move away, or to at least consider moving away. Many would only move to the extent that they were still living in what they feel is geographically part of Taranto because of a sense of belonging best encapsulated by the common statement, “*Il Tarantino si sente Tarantino* – the Tarantino feels Tarantino.” The participants retain a strong sense of belonging as ‘Tarantini’ (Taranto people), and an entitlement and a moral obligation to remain in the city. This paradox of residents who view pollution as a ‘monster’, and yet remain attached to the city is bridged through the culturally resonant expression of emotional distance. The relationship between this sense of belonging and experiences of risk requires further inquiry, for belonging and feeling safe could be more than a counter-narrative to risk-centred narratives of pollution in Taranto. The sense of attachment to place underlined by previous studies (d’Ovidio, 2021; Jokela-Pansini and Militz, 2022) and manifested by study participants as a way of creating safety could even be reinforced as a response to environmental narratives centred around pollution. This could shed new light on more ambivalent aspects of chronically contaminated areas, understanding how these modes of risk and safety creation could be connected to the identity of residents of chronically polluted areas.

Creating safety is, therefore, an affective concept grounded in resilience that strengthens residents’ relations with their environment through emotions, feelings, and sensory experiences in response to perceptions of industrial risk (Knight and Stewart, 2016; Duffy, Gallagher and Waitt, 2019). Such attachments and insights, alongside more conventional accounts of environmental harms (Checker, 2007; Pulido and De Lara, 2018; Givens, Huang and Jorgenson, 2019), lay the groundwork for not only resilience but hope for future restoration and recovery.

## Figures and Tables

**Table 1:** Background characteristics of the respondents.

	N	%
**Gender**		
Female	96	53
Male	83	46
Other	3	1
**Age**		
18–34	43	24
35–64	110	61
65+	28	15
**Education**		
Primary level	7	4
Secondary level	24	13
High school	80	43
University degree	73	40
**Has children**	90	51
**Employment status**		
Employed	78	46
Self-employed	33	19
Unemployed	24	14
Student	8	5
Retired	27	16
**Occupational category (profession)**		
Employer	13	13
Management	20	20
Employed	56	56
Worker	10	10
**Income**		
0-10 000	58	37
10 000–20 000	34	22
20 000–35 000	44	28
35 000–60 000	14	9
60 000+	5	3

**Table 2:** Survey questions.

l.Are there any problems in your neighbourhood that have a negative impact on your quality of life? If yes; could you give us one or more examples?
2.If you think about your quality of life, which of the following is most important to you? Improving the local environment Decreasing crime Improving access to services (health, education) None of the above Prefer not to say Other, please elaborate
3.How would you assess the environmental situation in your neighbourhood? Very good, good, nor good nor bad, bad, very bad
4.How would you assess the environmental situation in Taranto? Very good, good, nor good nor bad, bad, very bad
5.How would you assess the environmental situation in Apulia? Very good, good, nor good nor bad, bad, very bad
6.What do you think are the biggest environmental problems in your neighbourhood? Individual people’s improper waste disposal The city’s improper waste disposal Illegal landfills Car fumes Dust from industries Odours from industries The oil refinery Ex-ILVA steel plant Water pollution Atmospheric pollution Pesticides Mould Humidity Crime I don’t think there are any problems in my neighbourhood Prefer not to say
7.Do you think there is an elevated risk of the following health issues in your neighbourhood? Respiratory problems Irritation in eyes Reproductive problems Cardiovascular problems Cancer Other None Prefer not to say
8.Do you worry that environmental pollution has an effect on your health/your children’s health/other family members? Yes/No
9.If you worry about environmental pollution, has this had an impact on your mental health in any of the following ways? Depression Anxiety Anger Frustration Resignation Helplessness
10.Are there any other ways you feel the pollution influences your everyday life?
11.How do you mitigate exposure to pollution?
12.Are there any other things you do to protect yourself and your family from pollution?
13.In the past years, have you considered or already done the following: Moving away from your neighbourhood because of pollution Moving away from Taranto because of pollution
14.How do you think environmental pollution could be reduced?
15.Who do you think is responsible for protecting residents from/taking action on pollution?

## Data Availability

The data that has been used is confidential.
